# Efficacy and safety of solifenacin succinate in Korean patients with overactive bladder: a randomised, prospective, double-blind, multicentre study

**DOI:** 10.1111/j.1742-1241.2008.01898.x

**Published:** 2008-11

**Authors:** M-S Choo, J Z Lee, J B Lee, Y-H Kim, H C Jung, K-S Lee, J C Kim, J T Seo, J-S Paick, H-J Kim, Y G Na, J G Lee

**Affiliations:** 1 Department of Urology, Asan Medical Center, University of Ulsan College of MedicineSeoul, Korea; 2 Department of Urology, College of Medicine, Pusan National UniversityBusan, Korea; 3 Department of Urology, National Medical CenterSeoul, Korea; 4 Department of Urology, Soonchunhyang University Bucheon HospitalBucheon, Korea; 5 Department of Urology, Yeungnam University HospitalDaegu, Korea; 6 Department of Urology, Samsung Medical Center, Sungkyunkwan University School of MedicineSeoul, Korea; 7 Department of Urology, College of Medicine, The Catholic University of KoreaSeoul, Korea; 8 Department of Urology, Jeil Hospital, Kwandong University College of MedicineSeoul, Korea; 9 Department of Urology, Seoul National University College of MedicineSeoul Korea; 10 Department of Urology, Dankook University College of MedicineCheonan, Korea; 11 Department of Urology, Chungnam National University College of MedicineDaejeon, Korea; 12 Department of Urology, College of Medicine, Korea UniversitySeoul, Korea

## Abstract

**Purpose:**

We assessed the efficacy and safety of solifenacin compared with tolterodine for treatment of overactive bladder (OAB) in Korean patients.

**Materials and methods:**

The study was randomised, double-blind, tolterodine-controlled trial in Korea. Patients had average frequency of ≥ 8 voids per 24 h and episodes of urgency or urgency incontinence ≥ 3 during 3-day voiding diary period. Patients were randomised to 12-week double-blind treatment with either tolterodine immediate release (IR) 2 mg twice daily (TOL4) or solifenacin 5 mg (SOL5) or 10 mg (SOL10) once daily. The outcome measure was mean change in daily micturition frequency, volume, daily frequency of urgency incontinence, urgency and nocturia from baseline to week 12. Quality of life was assessed using the King’s Health Questionnaire.

**Results:**

A total of 357 were randomised and 329 were evaluated for efficacy. All voiding parameters recorded in micturition diary improved after treatment in all three groups. Mean changes in volume voided were 19.30 ml (26.69%) in TOL4, 30.37 ml (25.89%) in SOL5 and 37.12 ml (33.36%) in SOL10 group (p = 0.03). Speed of onset of SOL10 efficacy on urgency incontinence was faster than that of SOL5 and TOL4. Quality of life improved in all three groups. Dry mouth was the most common adverse event; its incidence was the lowest in SOL5 group (7.63%, compared with 19.49% and 18.64% in SOL10 and TOL4 groups respectively).

**Conclusions:**

Solifenacin succinate 5 and 10 mg once daily improve OAB symptoms with acceptable tolerability levels compared with tolterodine IR 4 mg. Solifenacin 5 mg is a recommended starting dose in Korean patients with OAB.

**What's known**

- Pharmacological management using antimuscarinic agents remains the mainstay of therapy and effectively reduces OAB symptoms.
- A new-generation antimuscarinic agent, solifenacin succinate is a once-daily oral agent that shows apparent functional selectivity for the bladder over other organs in animal models.
- There have been no published comparative data between solifenacin and tolterodine in Asian individuals.

**What's new**

- Compared with tolterodine, the treatment of solifenacin induces the improvement in OAB symptom and quality of life in Korean patients with OAB.
- This is the first report to compare efficacy and safety of solifenacin succinate with tolterodine in Asian people with OAB. Solifenacin succinate 5 and 10 mg improve OAB symptoms and solifenacin 5 mg is a recommended starting dose.

## Introduction

Overactive bladder (OAB) syndrome has been defined by the International Continence Society as ‘a syndrome comprising the symptoms of urgency, with or without urgency incontinence, usually with frequency and nocturia’ (1). Although OAB is not life-threatening, it significantly impairs patients’ quality of life (2).

Pharmacological management using antimuscarinic agents remains the mainstay of therapy and effectively reduces OAB symptoms (3). However, despite the proven efficacy of such agents, their tolerability may be limited by adverse events (AEs), mostly dry mouth (4). In fact, these adverse events often lead to poor compliance and discontinuation of therapy (5). OAB follows a chronic course requiring long-term therapy, so new agents with proven efficacy and better tolerability are required.

A new-generation antimuscarinic agent, solifenacin succinate (Vesicare^®^; Astellas Pharma Co. Ltd, Tokyo, Japan), is a once-daily oral agent that shows apparent functional selectivity for the bladder over other organs in animal models (6). In phase 2 trials, symptomatic OAB patients have shown statistically significant reductions in voiding frequency and a significant increase in volume voided per void with doses of 5, 10 and 20 mg once daily (7). In large phase 3 trials, solifenacin 5 or 10 mg once daily demonstrated good efficacy and tolerability profiles, and these improvements were maintained in a 40-week open-label extension study of two of the phase 3 trials (8–12).

Until now there have been no published comparative data between solifenacin and tolterodine in Asian individuals. In this study, we compared the efficacy and tolerability of solifenacin 5 and 10 mg once daily and tolterodine 2 mg twice daily in patients with symptoms of OAB.

## Patients and methods

This multicentre, randomised, double-blind, tolterodine-controlled phase 3 trial was conducted at 16 university hospitals in Korea. The procedures of this study complied with the guidelines provided by the Declaration of Helsinki and were in accordance with the International Conference on Harmonization Good Clinical Practice guidelines. The study protocol was approved by the institutional review board at each study site.

The objectives of the study were to assess efficacy and tolerability of 12-week treatment with solifenacin 5 mg (SOL5) and 10 mg (SOL10) compared with 12-week tolterodine immediate-release 4 mg (TOL4) in patients with OAB. At the time of study, the tolterodine extended-release (ER) formulation was not available in Korea. The primary outcome measure was change in the mean daily micturition frequency from baseline to end-point. The secondary outcome measures were changes from baseline in the mean micturition volume per voiding, mean daily (24 h) urgency incontinence frequency, mean daily number of urgency episodes, mean daily number of nocturia episodes according to the micturition diary and quality of life as assessed by the King’s Health Questionnaire (13,14). Safety was assessed by AEs, laboratory tests, vital signs and postvoid residual (PVR) volumes as measured by bladder scans.

Eligible patients visited the investigational sites for screening (visit 1), at the end of the placebo run-in period (visit 2) and at weeks 4, 8 and 12 of the double-blind period (visits 3, 4 and 5). Men and women aged ≥ 18 years with symptoms of OAB for ≥ 3 months were eligible for screening and study enrolment. To be eligible for randomisation after the 2-week placebo run-in period, patients had to have had an average frequency of ≥ 8 voids per 24 h and have experienced at least three episodes of urgency or three episodes of urgency incontinence during the 3-day voiding diary period. Exclusion criteria included clinically significant bladder outlet obstruction, a PVR volume of > 200 ml, incontinence for which stress was determined to be the predominant factor, presence of a neurological cause for detrusor muscle overactivity, evidence of urinary tract infection or bladder stones, previous pelvic irradiation, or previous or current malignant disease in the pelvic organs, any medical condition contraindicating the use of antimuscarinic medication (including narrow-angle glaucoma and urinary or gastric retention), non-pharmacological treatment for OAB including electrostimulation therapy or start of a bladder training programme during the 2 weeks before or during the study, diabetic neuropathy, use of drugs intended to treat incontinence, use of any drugs with cholinergic or anticholinergic side effects and participation in a clinical trial within 30 days before study entry. Women of childbearing potential who were pregnant or nursing, intending to become pregnant during the study, or who were not using reliable contraceptive methods were ineligible.

At an initial screening visit, the patients provided a medical history and underwent a physical examination, bladder scan for PVR volume, blood and urinalysis and an electrocardiogram. Eligible patients received placebo twice daily (morning and evening) over a 2-week run-in period. During the 3 days before the next visit, patients recorded episodes of urgency and urgency incontinence, the times of voiding and volumes voided per void in a voiding diary. After the run-in period, eligible patients were randomised equally to 12-week double-blind treatment with TOL4, SOL5 or SOL10. To maintain blinding, all patients continued to take medication twice daily (using placebo tablets and capsules as necessary) during the 12-week treatment period. Patients were regarded compliant if they had taken at least 70% of the required study medication.

Adverse events were evaluated for individuals receiving at least one dose of active study medication. At each visit, any AE reported in response to general and non-specific questioning by the investigator, or self-reported by the patient was recorded with the severity and likely causality to study medication. Safety assessments at weeks 4, 8 and 12 included vital signs, physical examinations and AE recordings. Laboratory screening and electrocardiogram scans were repeated at the end of the study. The PVR volume was assessed by bladder scanning (the same method was used for each patient) at the start and finish of the 12-week treatment period.

Efficacy comparison among treatment groups used the hierarchical (step-down) test procedure. ANOVA was used to compare the change from baseline to end-point in the mean daily micturition frequency between SOL10 and TOL4 (a non-inferiority test using 95% confidence interval). If SOL10 was found not to be inferior to TOL4, the hierarchical (step-down) test procedure was progressed to the second step. The second step analysis compared SOL5 and TOL4. The paired *t*-test was used to analyse the mean change from baseline to end-point, and the Stuart-Maxwell chi-square test, McNemar’s chi-square test, Mantel-Haenszel chi-square test and Fisher’s exact test were used to analyse deviation from the normal range. For subject withdrawal, data available at the point of withdrawal were analysed. Missing data were accepted as such. Nonetheless, data analysis with the last observation carried forward (LOCF) method was performed and presented for efficacy analysis. With the LOCF analysis, the missing values are replaced by the last observed value of that variable. The statistical analyses of the AEs were done by Fisher’s exact test.

## Results

**Figure 1:**
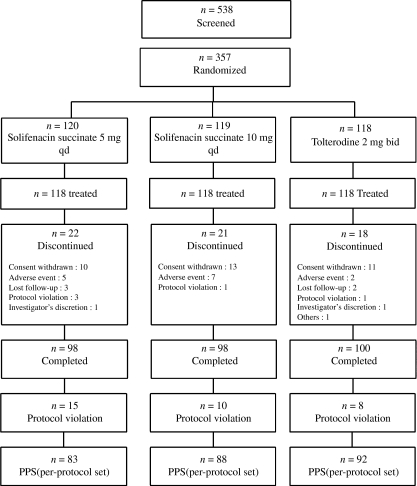
Patient disposition

A total of 538 patients with OAB were screened, of whom 357 were randomised and 354 were treated. The efficacy analysis included all randomised patients who had efficacy data available from the baseline and at least one on-treatment visit (329 patients). In all, 61 patients (17.1%) discontinued the study before completion; this was because of AEs for 14 patients (3.9%), withdrawal of consent for 34 patients (9.5%) and other reasons for 13 patients (3.7%). A total of 81.7% of patients in the SOL5 group, 82.4% in the SOL10 group and 84.8% in the TOL4 group had their final (end-point) efficacy evaluation at week 12 (Figure 1).

Baseline characteristics and demographics were similar among all treatment groups as shown in Table 1. The mean age of all patients was 52.65–53.07 years. Of all patients, 24.32–30.36% had received previous drug treatment for OAB and 42.06–52.25% reported urgency incontinence. At baseline, there were no differences in voiding parameters among the three groups (p > 0.05).

**Table 1:** Patient demographics at baseline

	Solifenacin succinate			
Characteristic	5 mg qd (*N* = 107)	10 mg qd (*N* = 111)	Tolterodine 2 mg bid (*N* = 111)	p-value
**Age (years)**				
Mean	53.07	52.65	53.05	0.9568*
SD	10.52	12.71	12.19	
Median	52.00	54.00	53.00	
Range	25.00–86.00	23.00–80.00	24.00–78.00	
**Gender**				
Male	17 (15.39)	28 (25.23)	23 (20.72)	0.2349†
Female	90 (84.11)	83 (74.77)	88 (79.28)	
**Weight (kg)**				
Mean	59.62	60.18	59.09	0.6722*
SD	9.85	8.92	8.44	
Median	59.00	59.00	58.00	
Range	42.00–110.00	43.00–85.00	42.00–81.00	
**Height (cm)**				
Mean	157.93	159.83	158.64	0.1364*
SD	6.62	7.57	7.12	
Median	158.00	159.00	158.00	
Range	139.00–173.00	140.00–181.00	141.30–182.00	
**Pulse (time/min)**				
Mean	74.13	73.55	72.18	0.3342*
SD	10.97	9.96	9.00	
Median	74.00	72.00	72.00	
Range	48.00–120.00	52.00–109.00	52.00–103.00	
**Diastolic BP (mmHg)**				
Mean	123.82	120.70	124.77	0.2053*
SD	17.83	16.96	18.50	
Median	122.00	120.00	123.00	
Range	70.00–172.00	88.00–177.00	90.00–174.00	
**Systolic BP (mmHg)**				
Mean	76.71	74.77	76.28	0.4327*
SD	11.99	10.83	12.18	
Median	79.00	74.00	74.00	
Range	40.00–108.00	51.00–106.00	50.00–114.00	

* ANOVA.

† Chi-square test. BP, blood pressure.

**Figure 2:**
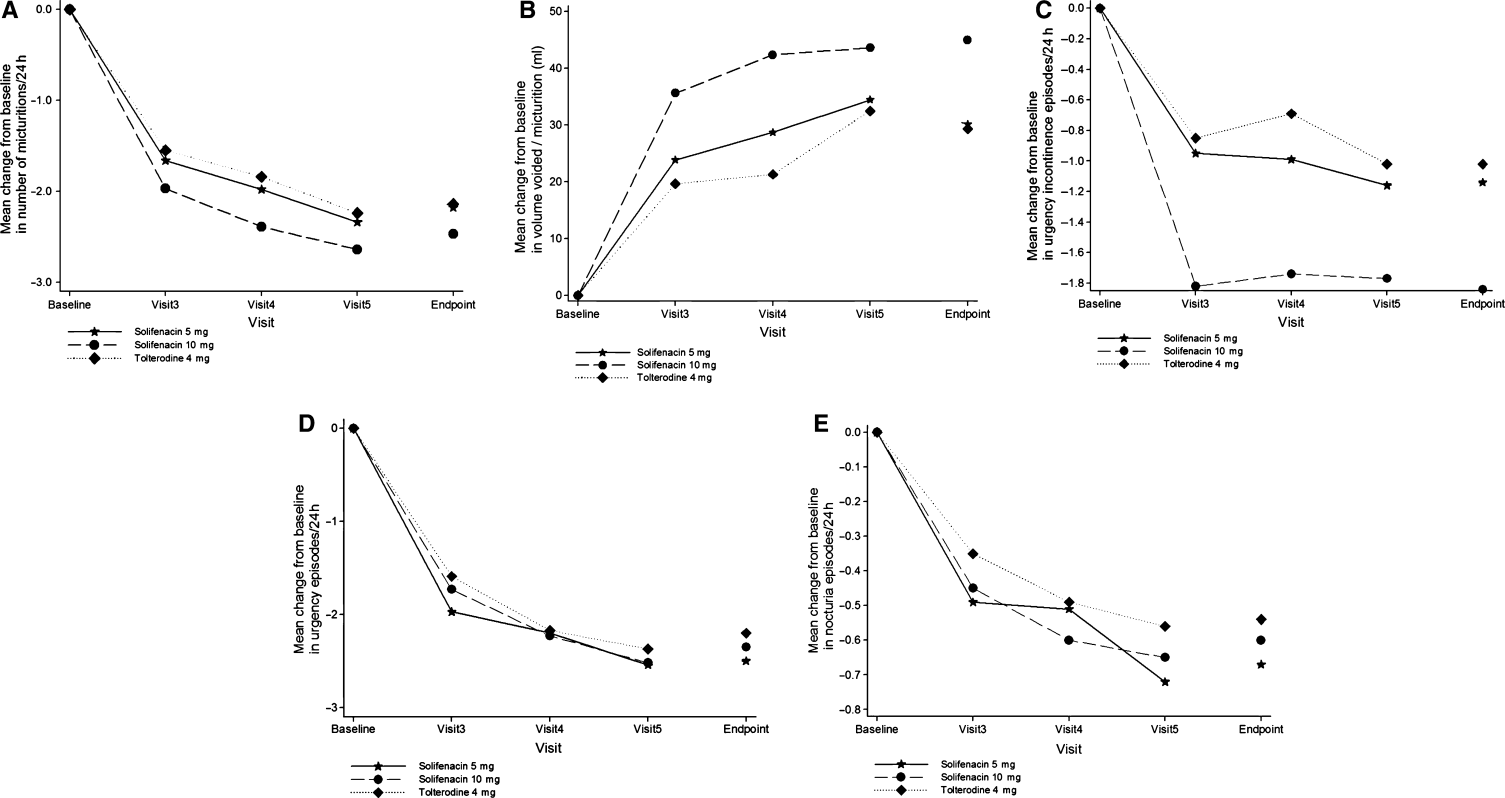
(A) Changes in the mean daily micturition frequency. (B) The mean micturition volume per voiding (ml). (C) The mean daily urgency incontinence frequency. (D) The mean daily number of urgency episodes. (E) The mean number of nocturia episodes

Table 2 shows the mean changes in all efficacy variables from baseline to end-point in the three groups. After the 12-week treatment, these parameters improved. The improvement rate was the greatest in the SOL10 group, followed by the SOL5 and TOL4 groups (Figure 2). A non-inferiority test showed that the lower limit of the 95% confidence interval was −0.25, exceeding the limit of non-inferiority of −1. It showed that SOL10 was not inferior to TOL4, so the second step analysis was performed to compare SOL5 and TOL4. This showed that the lower limit of the 95% confidence interval was −0.49, exceeding the limit of non-inferiority of −1. Therefore, we also showed that SOL5 was non-inferior to TOL4.

**Table 2:** The mean changes in efficacy variables with solifenacin and tolterodine from baseline to end-point

	Solifenacin succinate		
	5 mg qd	l0 mg qd	Tolterodine 2 mg bid
**Mean daily micturition frequency**			
Baseline	11.26 ± 2.78	11.19 ± 2.82	11.44 ± 2.81
Visit 3	9.60 ± 2.46	9.21 ± 2.46	9.88 ± 2.48
Visit 4	9.37 ± 2.87	8.83 ± 2.63	9.76 ± 2.63
Visit 5	9.01 ± 2.70	8.59 ± 2.70	9.23 ± 2.64
End-point	9.08 ± 2.58	8.71 ± 2.73	9.30 ± 2.61
End-point baseline (p = 0.78)	−2.18 (−18.29%)	−2.47 (−20.95%)	−2.14 (−16.90%)
**Mean micturition volume per voiding**			
Baseline	147.22 ± 51.11	154.03 ± 49.77	147.74 ± 49.06
Visit 3	171.09 ± 62.77	189.63 ± 63.24	167.86 ± 53.05
Visit 4	175.44 ± 62.21	194.56 ± 75.21	165.35 ± 58.61
Visit 5	181.11 ± 59.44	196.98 ± 69.96	177.93 ± 68.26
End-point	177.38 ± 59.08	198.99 ± 73.58	177.08 ± 66.41
End-point baseline (p = 0.53)	30.16 (25.89%)	44.97 (33.36%)	29.34 (26.69%)
**Mean daily urgency incontinence frequency**			
Baseline	1.92 ± 2.19	2.58 ± 2.91	1.74 ± 1.55
Visit 3	0.97 ± 1.49	0.76 ± 1.10	0.90 ± 1.16
Visit 4	0.95 ± 1.81	0.77 ± 1.17	1.03 ± 1.41
Visit 5	0.78 ± 1.76	0.72 ± 1.51	0.67 ± 1.16
End-point	0.78 ± 1.74	0.75 ± 1.49	0.72 ± 1.18
End-point baseline (p = 0.14)	−1.14 (−59.38%)	−1.84 (−57.60%)	−1.02 (−52.30%)
**Mean daily number of urgency episodes**			
Baseline	4.29 ± 3.45	3.81 ± 3.04	3.89 ± 3.12
Visit 3	2.32 ± 3.00	2.09 ± 2.49	2.32 ± 2.86
Visit 4	2.11 ± 2.74	1.72 ± 2.40	1.83 ± 2.54
Visit 5	1.77 ± 2.74	1.42 ± 2.21	1.60 ± 2.88
End-point	1.79 ± 2.66	1.47 ± 2.34	1.68 ± 3.02
End-point baseline (p = 0.51)	−2.50 (−57.72%)	−2.35 (−63.50%)	−2.20 (−55.43%)
**Mean number of nocturia episodes**			
Baseline	1.82 ± 1.27	1.73 ± 1.07	1.77 ± 1.00
Visit 3	1.34 ± 1.14	1.28 ± 0.99	1.40 ± 0.96
Visit 4	1.31 ± 1.19	1.11 ± 0.98	1.25 ± 1.02
Visit 5	1.13 ± 1.12	1.07 ± 0.94	1.17 ± 1.09
End-point	1.15 ± 1.11	1.14 ± 1.01	1.23 ± 1.13
End point baseline (p = 0.85)	−0.67 (−31.41%)	−0.60 (−28.88%)	−0.54 (−24.85%)

The SOL10 group had a significantly increased micturition volume per voiding compared with TOL4 group (p = 0.04). Urgency incontinence episodes dramatically decreased in SOL10 group at visit 3 (4 week) compared with other groups although there was no statistical significance at end-point.

Subgroup analysis showed that in patients with dry OAB, the changes in mean daily micturition frequency from baseline were −2.92 in the SOL10 group and −1.85 in the TOL4 group (p = 0.019). In male patients with wet OAB, the changes in mean daily urgency incontinence frequency from baseline were −2.92 in the SOL10 group and −1.85 in the TOL4 group (p = 0.037).

Quality of life improved in all three groups using the King’s Health Questionnaire. But both solifenacin treatments (10 and 5 mg) produced greater improvements from baseline compared with TOL4, although these results were not statistically significant (Table 3).

**Table 3:** Changes in the quality of life, as assessed by the King’s Health Questionnaire (ANOVA)

		Solifenacin succinate	
		5 mg qd (*N* = 107)	10 mg qd (*N* = 111)
Quality of life Domain	Tolterodine 2 mg bid (*N* = 111) Adjusted mean change from baseline (*N*)	Adjusted mean change from baseline (*N*), estimated difference to tolterodine, p-value hierarchical test	
General health perception	−1.96 (110)	−3.28 (107)	−2.33 (111)
		−1.32	−0.37
		–	0.9827
Incontinence impact	−12.10 (110)	−13.60 (107)	−15.94 (111)
		−1.50	−3.84
		–	0.2864
Role limitations	−19.47 (107)	−18.44 (105)	−23.75 (111)
		1.03	−4.28
		–	0.4160
Physical limitations	−20.39 (107)	−20.97 (105)	−25.26 (111)
		−0.58	−4.87
		–	−0.2815
Social limitations	−18.77 (103)	−13.95 (105)	−23.40 (110)
		4.83	−4.62
		–	0.5296
Personal relationships	−7.78 (67)	−9.31 (70)	−7.08 (68)
		−1.53	0.70
		–	0.9792
Emotions	−15.34 (105)	−16.86 (107)	−20.37 (110)
		−1.52	−5.03
		–	0.2369
Sleep/energy	−13.66 (107)	−15.68 (107)	−18.75 (110)
		−2.02	−5.09
		–	0.1849
Severity measure	−9.87 (99)	−12.77 (105)	−13.76 (106)
		−2.90	−3.89
		–	0.2420
Symptom severity	−3.48 (110)	−3.36 (107)	−4.03 (111)
		0.12	−0.55
		–	0.4813

The discontinuation rates because of AEs were low and were similar for the three treatment arms. The percentages of patients discontinuing treatment owing to an AE were 4.24% (5/118), 5.93% (7/118) and 1.69% (2/118) in the SOL5, SOL10 and TOL4 groups, respectively (p = 0.28). The incidences of AEs (≥ 2%) after medication are listed in Table 4. Among the treatment groups, the incidence of the most common AE, dry mouth, was lowest in the SOL5 group (7.63%, compared with 19.49% and 18.64% in the SOL10 and TOL4 groups, respectively; p = 0.01). Constipation, which was mainly mild or moderate in all groups, was reported in 6.78%, 14.41%, 2.54% of patients in the SOL5, SOL10 and TOL4 groups, respectively (p = 0.003). The incidence of constipation was not statistically different between SOL5 and TOL4 group (p = 0.215) but different between SOL10 and TOL4 group (p = 0.002). Blurred vision (mild in most cases) was reported in 13.56%, 16.95% and 10.17% of patients in the SOL5, SOL10 and TOL4 groups, respectively (p = 0.335).

**Table 4:** Incidence rates of adverse events after medication

	Solifenacin succinate		
Adverse events*	5 mg qd (*N*= 118), *n* (%)	10 mg qd (*N*= 118), *n* (%)	Tolterodine 2 mg bid (*N* = 118), *n* (%)
Gastrointestinal disorders	22 (18.64)	42 (35.59)	30 (25.42)
Dry mouth	9 (7.63)	23 (19.49)	22 (18.64)
Constipation	8 (6.78)	17 (14.41)	3 (2.54)
Dyspepsia	3 (2.54)	4 (3.39)	2 (1.69)
Abdominal pain upper	2 (1.69)	4 (3.39)	1 (0.85)
Eye disorders	17 (14.41)	25 (21.19)	18 (15.25)
Vision blurred	16 (13.56)	19 (16.10)	12 (10.17)
Dry eye NOS	0 (0.00)	1 (0.85)	3 (2.54)
Renal and urinary disorders	6 (5.08)	10 (8.47)	14 (11.86)
Difficulty in micturition	1 (0.85)	6 (5.08)	6 (5.08)
Urine flow decreased	3 (2.54)	3 (2.54)	3 (2.54)
Vesical tenesmus	1 (0.85)	1 (0.85)	3 (2.54)
Infections and infestations	7 (5.93)	5 (4.24)	6 (5.08)
Cystitis NOS	3 (2.54)	2 (1.69)	2 (1.69)
Nasopharyngitis	1 (0.85)	1 (0.85)	3 (2.54)
Nervous system disorders	2 (1.69)	3 (2.54)	3 (2.54)
Injury, poisoning and procedural complications	0 (0.00)	5 (4.24)	2 (1.69)
General disorders and administration site conditions	0 (0.00)	1 (0.85)	4 (3.39)
Skin and subcutaneous tissue disorders	1 (0.85)	3 (2.54)	1 (0.85)

*Dictionary: MedDRA 5.0 (incidence of AEs ≥ 2.0%). NOS, not otherwise specified; AEs, adverse events.

There were no clinically relevant changes in vital signs, physical examination findings, laboratory values or electrocardiogram results. PVR volumes increased in all groups but they were not clinically significant (Table 5). There were no differences of PVR according to the treatment groups and gender (p = 0.826, 0.227), although male patients had more increased PVR than female.

**Table 5:** The mean changes in postvoid residual urine volume from baseline to end-point

	Solifenacin succinate				
	5 mg qd (*N* = 118)		10 mg qd (*N* = 118)		Tolterodine 2 mg bid (*N* = 118)	
Postvoid residual volume (ml)		Male	Female	Male	Female	Male	Female
Baseline	*N*	22	96	30	87	23	95
	Mean (SD)	25.32 (31.18)	22.77 (33.45)	30.97 (28.90)	30.17 (37.89)	27.09 (28.30)	21.99 (36.50)
	Range	0.00–99.00	0.00–195.00	0.00–97.00	0.00–173.00	0.00–90.00	0.00–247.00
End of study	*N*	19	91	29	83	22	86
	Mean (SD)	37.68 56.39)	29.93 (39.76)	41.97 (61.68)	35.14 (60.25)	34.04 (45.10)	26.78 (35.80)
	Range	0.00–241.00	0.00–197.00	0.00–287.00	0.00–399.00	0.00–175.00	0.00–184.00
Change	*N*	19	91	29	82	22	86
	Mean (SD)	10.26 (48.59)	7.95 (39.26)	9.93 (54.32)	2.76 (49.31)	6.31 (39.55)	4.37 (29.42)

ANCOVA, treatment drug (p = 0.8257), gender (p = 0.2266).

## Discussion

Overactive bladder substantially compromises patients’ quality of life. Therefore, effective treatment of OAB must result in a meaningful reduction in all of these symptoms. Currently, available antimuscarinic agents effectively relieve symptoms and are comparable in having similar efficacies (15). Solifenacin is a potent M3-selective receptor antagonist with high functional selectivity for the urinary bladder.

This randomised tolterodine-controlled clinical trial evaluated the clinical efficacy of different doses of solifenacin in patients with OAB symptoms for at least 3 months. In the evaluation of primary efficacy outcome measure at end-point, both solifenacin doses were associated with significant improvements in the mean daily micturition frequency compared with baseline; the micturition frequency was reduced to about eight voids per day, and these results were not inferior to those achieved with TOL4. The secondary efficacy variable, mean micturition volume per voiding, was also significantly improved with all three treatment groups, showing that the two solifenacin doses were not inferior to TOL4; indeed, the increase was more significant for the SOL10 group than for the TOL4 group. The mean volume voided per micturition has been used in randomised clinical trials to assess the efficacy of medical treatment. The volume voided per micturition may be less affected by placebo than the number of micturitions per day and an increase in voided volume per micturition indicates that the bladder capacity is greater at the time of micturition (16). Therefore, significant increases in volume per micturition in the SOL10 group compared with the SOL5 and TOL4 groups are meaningful. The magnitudes of changes in mean daily urgency, urgency incontinence frequency and nocturia demonstrated that solifenacin has a similar efficacy as tolterodine. The results of a European double-blind tolterodine-controlled trial including large numbers of randomised patients (1081 patients) are quite different from our data (9). Compared with tolterodine 2 mg twice daily, the changes in efficacy parameters such as episodes of urgency and urgency incontinence were more marked in solofenacin 5 and 10 mg groups.

According to the present study, SOL5 is an appropriate initial therapy for OAB patients in Korea. Although the changes in efficacy variables are greater with SOL10 than SOL5, these differences are not statistically significant, with the exception of mean micturition volume per voiding. In a prospective, randomised, double-blind study in Japan comparing placebo, SOL5, SOL10 and propiverine hydrochloride 20 mg, there were also no significant differences in the primary efficacy variable, mean number of voids per 24 h, between the SOL5 and SOL10 groups (17). In the same study, among patients who had urgency incontinence at baseline, 56.2% and 59.6% of patients in the SOL5 and SOL10 groups, respectively, achieved continence at study end-point. Likewise, 61.4%, 56.4% and 57.7% of incontinence patients treated with SOL5, SOL10 and TOL4, respectively, achieved continence at study end-point in the present study (data not shown).

Antimuscarinic agents have unwanted effects on other organs, the most common of which is dry mouth, and these may interfere with patient compliance and thus overall treatment efficacy (5). Dry mouth is common even with newer formulations such as oxybutynin ER (18) and tolterodine ER (19). Thus, there is a need for more-tolerable therapeutic options with proper symptom relief. Both doses of solifenacin were well tolerated by patients, with only a few discontinuations caused by AEs. Importantly, the incidence of dry mouth was 7.6% in the SOL5 group and 19.5% in the SOL10 group, compared with 18.6% in the TOL4 group. In the European study, dry mouth was the most common AE but was well tolerated and reported in 14.0%, 21.3% and 18.6% of patients in the SOL5, SOL10 and TOL4 groups, respectively (9). Although constipation and blurred vision occurred more often in patients treated with solifenacin than in those treated with tolterodine, most of these AEs were mild to moderate and rarely led to discontinuation of therapy.

In isolated cell preparations from rats and monkeys, solifenacin showed significantly more selectivity for the bladder over salivary gland tissue compared with tolterodine (20). The results of the present study are consistent with pharmacodynamic observations in phase 1 studies that compared with placebo, SOL5 has similar effects on salivary secretion and on the visual near point (21). The clinical effectiveness of SOL5 in terms of both tolerability and efficacy is clear from the present data, as it was associated with the most-favourable therapeutic index in the present study. These data are consistent with preclinical data indicating the relative selectivity of solifenacin for the bladder over the salivary gland, particularly the low incidence of dry mouth.

Furthermore, solifenacin significantly improved the Kings Health Questionnaire scores from baseline, demonstrating statistically significant improvements of patient quality of life. The mean change in Kings Health Questionnaire scores from baseline was greater in both the SOL5 and SOL10 groups compared with the TOL4 group, although the difference was not significant. Kelleher et al. (22) reported quality of life data, assessed by the Kings Health Questionnaire from two phase 3, 12-week studies (1984 patients) and long-term extensions of these studies (1637 patients) in which patients received solifenacin for up to an additional 40 weeks. Pooled data from the two studies showed that compared with placebo, solifenacin was associated with significant (p < 0.05) improvements in nine of 10 quality of life domains. About two-thirds of this overall improvement with solifenacin occurred during the original 12-week study, with an additional one-third reported during the extension period.

In conclusion, after the 12-week treatment of solifenacin 5 and 10 mg compared with tolterodine ER 4 mg, all the OAB symptoms and quality of life improved. The improvement rate was the greatest in the SOL10 group, followed by the SOL5 and TOL4 groups although micturition volume per voiding only had a statistical difference. The discontinuation rates caused by AEs were low and were similar for the three treatment arms. The dry mouth was the lowest in the SOL5 group. Solifenacin 5 mg is a recommended starting dose in Korean patients with OAB.
